# Global Carriage of Antimicrobial Resistance Genes and Virulence Factors Among Physicians in Cirrhosis Care

**DOI:** 10.21203/rs.3.rs-11319770/v1

**Published:** 2026-11-01

**Authors:** Andrew J Sommer, Masoumeh Sikaroodi, Andrew Fagan, Brian C Davis, Alexander Khoruts, Patrick M Gillevet, Jasmohan S Bajaj

**Affiliations:** University of Minnesota; George Mason University; Virginia Commonwealth University and Richmond VA Medical Center; Virginia Commonwealth University and Richmond VA Medical Center; University of Minnesota; George Mason University; Virginia Commonwealth University and Richmond VA Medical Center

## Abstract

Cirrhosis patients are at high risk for drug-resistant infections, which may be transmitted between patients and healthcare providers. In this study, we used shotgun metagenomics to characterize the fecal microbiome of physicians in cirrhosis care (n = 55) across 9 countries. Our results indicate physicians from low- or middle-income countries carry greater antimicrobial resistance gene (ARG) diversity corresponding to increased taxonomic richness. Physicians carried a low prevalence of clinically relevant ARGs or virulence factors (VFs), regardless of country of practice or patient exposure. Further comparison of fecal microbiomes between a subset of cirrhosis patients and physicians’ fecal samples collected from the same medical facility in the United States indicated that cirrhosis patients carried a far greater burden of high-risk VFs and ARGs. Collectively, this study provides evidence that most physicians in cirrhosis care are unlikely to be a primary reservoir of drug-resistant pathogens, although further surveillance of healthcare workers is warranted.

## INTRODUCTION

The emergence of drug-resistant pathogens represents a growing threat to global public health, with recent estimates indicating that bacterial antimicrobial resistant infections are responsible for over one million deaths annually.^1^ Patients with liver cirrhosis represent a particularly vulnerable patient population due to alterations in gut microbiome composition and function, impaired systemic and gastrointestinal immune function, and repeated hospitalizations and exposures to hospital settings^2^. Infections caused by Enterobacteriaceae (*e.g. Escherichia coli, Klebsiella pneumoniae*), *Enterococcus*, and other opportunistic pathogens are associated with high mortality in cirrhosis.^3,4^ These factors could also create an environment conducive for the colonization of antimicrobial-resistant opportunistic pathogens.^5,6^ The dissemination of antimicrobial resistance genes (ARGs) within the gut microbiome of patients with cirrhosis, including vancomycin resistance genes and extended-spectrum β-lactamase (ESBL) genes, presents a significant challenge to the effective treatment of these life-threatening infections^7^.

Healthcare settings may serve as important reservoirs of ARGs, where frequent antibiotic exposure, close patient contact, and environmental contamination can facilitate ARG acquisition and transmission.^8^ Physicians and other healthcare professionals, who have substantial contact with patients, have been implicated as potential carriers of antimicrobial resistant organisms, although metagenomic studies on physician ARG carriage are limited. ^9-11^ Global differences in healthcare resources and practices, including patient exposure levels, background resistance rates, access to personal protective equipment, and infection control, may influence the acquisition and carriage of antimicrobial resistance determinants among healthcare personnel, underscoring the need to characterize ARG dissemination across diverse healthcare settings. These vary according to the country and center, specifically related to infections in cirrhosis^12,13^.

To address this gap, we performed shotgun metagenomic sequencing from stool samples collected from 55 physicians across 9 countries with varying degrees of cirrhosis patient exposure to identify global differences in ARG and virulence factor (VF) carriage. We further compared the ARGs and VFs between 8 physicians and 172 cirrhosis patients within the same facility to assess the potential for shared antimicrobial resistance and virulence factor profiles between physicians and cirrhosis patients.

## RESULTS

### Physician Cohort Characterization:

We enrolled 55 physicians (all were physicians) from 9 countries; 42 were from high income countries (HICs) practicing in Denmark (n = 1), Lithuania (n = 5), and USA (n = 36) while 13 were from low- and middle-income countries (LMICs) including those practicing in Brazil (n = 4), China (n = 1), India (n = 3), Mexico (n = 3), Thailand (n = 1), and Turkey (n = 1). Eight of the US-based physicians were physicians working at VCU and Richmond VA Medical Center. The remaining US-based physicians were from the West coast (n = 13), Northeast (n = 6), rest of the South (n = 5) and from the Midwest (n = 4). Most physicians were men and practicing in public hospital settings. Approximately half of the physicians practicing in HICs were born outside the current country of practice, and 42% reported recent trips outside country of practice. Inpatient cirrhosis patient exposure was similar between doctors practicing in HICs and LMICs. Six physicians in HICs and five in LMICs were classified as having high exposure to cirrhosis patients with resistant infections. Inpatient and outpatient cirrhosis patient exposure were similar but there was a trend towards higher resistant infections in patients seen by physicians in LMICs. A summary of physician demographics is included in the supplemental (**Table S1**).

### Physicians from LMICs carry a greater diversity of ARGs

We first evaluated overall carriage of ARGs in physician fecal metagenomes stratified by practicing country income level through read-based alignment against the ResFinder database. While no significant differences in total normalized ARG abundance were observed (Fig. 1A, Wilcoxon rank-sum test, p = 0.090), physicians practicing in LMICs carried a greater number of detected ARGs compared to physicians practicing in HICs (Fig. 1B, Wilcoxon rank-sum test, p < 0.001). A generalized linear model was fit to evaluate the association of observed ARG counts with species diversity and practicing-country income status (Fig. 1C, **Figure S1**). Overall ARG richness increased with increasing species richness (IRR = 1.001, p = 0.008), with physicians practicing in HICs harboring significantly lower ARG richness (IRR = 0.776, p = 0.001). Several ARGs showed a higher prevalence (Fisher’s exact test, nominal p-value < 0.05) in physicians practicing in LMICs, which included the *erm(G)*, blaACI-1, *cfxA3*, and *cat* genes (Fig. 1D), although none remained statistically significant after adjustment for multiple comparisons. MAG reconstruction indicated that the macrolide resistance gene *erm(G)* was encoded by multiple genera with the Bacteroidaceae and Lachnospiraceae families (**Figure S2**). The *cfxA3* β-lactam ARG was identified in several MAGs classified within the Bacteroidaceae (*Phocaeicola, Bacteroides*, and *Parabacteroides*) and Rikenellaceae (*Alistipes*) families; *bla*-ACI-1 was identified in MAGs classified as *Dialister* and *Acidaminococcus* (**Figure S3**).

Clinically relevant ARGs (e.g., β-lactamases commonly associated with Enterobacterales, vancomycin resistance genes, and quinolone resistance genes) were rare across physician metagenomes (Fig. 1E). The *bla*CTX ESBL gene, along with the *bla*CMY and *bla*DHA *AmpC* genes, were detected only in a single physician from a HIC, who reported a recent infection within the last three months. Vancomycin resistance genes (*VanHDX*) were detected in a different physician from a HIC. Quinolone resistance determinants (*qnr* genes) were identified from three physicians practicing in LMIC countries.

### Impact of patient exposure on ARG carriage by physicians:

Observed ARG counts trended higher in physicians with high levels of inpatient and outpatient exposure (Fig. 2A-B) and was significantly higher in physicians caring for patients with resistant infections (Fig. 2C, Wilcoxon rank-sum test, p = 0.018). No significant differences in the normalized total abundance of ARGs were observed between physicians stratified by high and low exposure to inpatients, outpatients, or those with known resistant infections (**Figure S4**, Wilcoxon rank-sum test, nominal p < 0.05). Several ARGs showed nominally significant differences in prevalence between exposure groups (Fisher’s exact test, nominal p < 0.05), including the *erm(X)* gene, which was more prevalent among doctors with high exposure across all three tested metrics (Fig. 2D). While the *erm(X)* gene was detected using read and contig-based approaches, no reconstructed MAGs carried this gene. The *tet(32)* was more prevalent in doctors with low inpatient exposure, which was detected on MAGs classified as *Mediterraneibacter, Blautia, Clostridium*, and *Eubacterium* (**Figure S5**). Similarly, *cfxA5* was more prevalent in doctors with low outpatient exposure and was detected on several Bacteroidaceae MAGs (**Figure S3**). Examination of ARG class prevalence indicated that indicated that tetracycline, macrolide-lincosamide (Mac-Lin), and aminoglycoside resistance genes were near ubiquitous across samples (Fig. 2E), which were broadly represented across a range of bacterial taxa (**Figures S2, S5, S6).**

### Global carriage of Enterobacteriaceae VFs by physicians:

We next examined if either exposure metrics or country of practice impact the carriage of key opportunistic pathogens in physicians. The prevalence of detectable *E. coli* was significantly higher in physicians practicing in LMICs compared to HICs (OR = 4.76, p = 0.029) (Fig. 3A). *K. pneumoniae* was found only in two doctors, including one doctor practicing in a HIC (Fig. 3B). No significant differences in *E. coli* prevalence were observed between low and high exposure metrics (**Figure S7**), although a visual increase was observed for all three metrics. A total of 16 physicians carried detectable Enterobacteriaceae VFs (Fig. 3C), which was lower than the overall prevalence of *E. coli*. Total normalized VF abundances varied substantially across these physicians (Fig. 3D). The physicians with a higher virulence factor burden included individuals from both income country types and reported variable levels of cirrhosis patient contact (Fig. 3D). Several notable VFs were detected among these physicians, including VFs associated with gut adhesion (*e.g.*, type 1 fimbriae, P fimbriae) and siderophore-mediated iron acquisition (*e.g.*, Enterobactin, Yersiniabactin, Aerobactin) (Fig. 3E).

### Cirrhosis patients carry a greater burden of clinically relevant ARGs and VFs:

We additionally analyzed a paired cohort of 8 physicians and 172 cirrhosis patients enrolled from the same medical facilities at VCU and Richmond VA. Of the 172 patients, 70 (41%) were compensated. Most (81%) were men, mean age was 59.5 ± 6 years and MELD score was 12 ± 5. Most patients had alcohol (36%) followed by viral hepatitis (33%), and MASH (19%) as the etiology. Almost half (54%) had HE, 35% had ascites and 12% had a variceal bleeding history. 37% were on rifaximin, 53% were on lactulose, 47% were on PPI, 23% on statins, and 3% on SBP prophylaxis at the time of stool collection.

The prevalence of *E. coli* was higher in both the compensated (64.3%) and decompensated cirrhosis patients (61.8%) compared to physicians (50%) (Fig. 4A). Furthermore, both *K. pneumoniae* and *Enterococcus* were detected in a subset of both compensated and decompensated cirrhosis patients while absent from all physician samples (Fig. 4B, **Figure S8**). Cirrhosis patients carried a substantially greater number of Enterobacteriaceae-associated VFs, with several cirrhosis patients encoding over 100 detectable VFs (Fig. 4C). Key clinically important VFs were unique to the cirrhosis patients, including genes related to iron acquisition, K1 capsule production, and fimbriae adhesion (Fig. 4E).

The overall diversity and normalized abundances of ARGs carried by cirrhosis patients and paired physician samples were not significantly different (p = 0.251) (Fig. 5AB), although physician samples maintained a higher species richness compared to decompensated patients (**Figure S9**). Despite no significant differences overall ARG counts, important clinically relevant ARGs, including vancomycin resistance genes, quinolone resistant genes, and Enterobacteriaceae-associated β-lactam resistance genes (e.g., *bla*-CTX, *bla*-SHV, *bla*-TEM), were unique to cirrhosis samples (Fig. 5C). A total of 5 β-lactam resistance genes were shared between the physician and patients, which were mainly variants of the *cfxA* gene family linked to commensal gut microbes (Fig. 5D, **Figure S3**). Screening of assembled MAGs within the dataset indicated that vancomycin resistance genes were detected on *Enterococcus* and *Blautia* MAGs while quinolone resistance genes were found on *Klebsiella, Enterobacter*, and *Escherichia* MAGs (**Figures S10-S11**).

Further comparison against all physician samples in the United States (n = 35) indicated that physicians harbored a greater diversity of ARGs than both compensated and decompensated cirrhosis patients (adjusted p = 0.0032 and p = 0.0012, respectively). After adjustment for multiple comparisons, we identified a total of 10 ARGs that showed significantly increased prevalence in physician samples compared to decompensated patients (Fisher’s exact test, adjusted p < 0.05) (**Figure S12)**. In contrast, we found that *tet(46)* was enriched in decompensated cirrhosis patients (p = 0.012), which was identified exclusively on *Streptococcus* MAGs (**Figure S5**).

We finally screened all assembled contigs within the dataset to identify the potential carriage of plasmid-borne ARGs within hospital settings. (**Figure S13**). The most prevalent plasmid replicon in both physician and cirrhosis patients was repUS52, which was detected on contigs co-encoding *tet(M)* resistant determinants. Several plasmid replicon types were detected on contigs co-encoding the narrow-spectrum β-lactamase genes *bla*-TEM, including IncFIC, IncFII, Inc1, and col(pHAD28) replicon sequences; however, plasmid-linked β-lactamase genes were only detected in a single physician (LMIC sample).

## DISCUSSION

In this multi-country analysis of physicians caring for patients with cirrhosis, we found that clinically relevant ARGs and VFs were only detected in a small proportion of participants. Although a subset of physicians harbored a high burden of Enterobacteriaceae VFs and associated ARGs, carriage of these genes largely did not track with patient exposure metrics or country of practice. Furthermore, paired analysis of physicians and patients from the same centers revealed that key clinically relevant ARGs and VFs were unique to patients, suggesting that the physicians were not a primary reservoir of drug-resistant pathogens within these facilities.

We hypothesized that frequent exposure to cirrhosis patients would drive elevated carriage of ARGs; however, high-concern clinical resistance determinants, including vancomycin resistance genes, quinolone resistance genes, and ESBLs were rare across physician samples. Although metagenomic data on healthcare workers remain limited, prior studies have indicated that ICU workers exhibit a higher burden of ARGs compared to other healthy individuals^9,10^. The prevalence of ESBL and vancomycin resistance carriage by physicians vary widely across culture-dependent studies^14-16^, which suggests that differences in geography, healthcare infrastructure, or localized transmission patterns may impact ARG occurrence. Although the physicians within this study did report on patient contact, the exposure could be lower than nurses, trainees, or other staff with prolonged and frequent patient interactions. Contact with environmental surfaces within the hospital (*e.g.*, beds, sinks) could also represent important reservoirs for ARGs^17^, which were not sampled in this study. A broader longitudinal sampling of environmental sources as well as physicians and nurses caring for the same patients could provide important insights into ARG transmission dynamics within hospitals.

The majority of detected ARGs in physician samples likely represented resistance determinants carried by commensal members of the gut microbiome, including strains of Lachnospiraceae and Bacteroidaceae. We observed an overall increase in ARG diversity among physicians from LMICs, which was significantly associated with greater species richness. This finding aligns with prior work demonstrating geographical variations in the gut microbiome and resistome associated with differences in lifestyle, diet, and socioeconomic status.^18,19^ Prior studies have also identified a higher overall burden of ARGs in lower income countries primarily attributed to differences in infectious disease exposure, antimicrobial usage, healthcare access, and sanitation. ^18^While the emergence of drug-resistant pathogens is expected to disproportionally impact lower-income regions^1^, our results highlight the need to disentangle true clinical resistance determinants from background resistome expansion driven by commensal diversity, especially among healthy individuals.

Understanding sources of transmission for *Enterococcus, E. coli*, and *K. pneumoniae* are of critical importance due to their role as leading causes of severe opportunistic infections in cirrhosis and other high risk patient populations. While *Enterococcus* and *K. pneumoniae* carriage was rare across all physician samples, *E. coli* was detected in 40.5% of physicians practicing in HICs and 76.9% of physicians practicing in LMICs. The increased prevalence in physicians from LMICs may reflect higher levels of environmental exposure to *E. coli* linked to differences in water and sanitation infrastructure.^20^ Notably, only a subset of physicians with *E. coli* also harbored detectable Enterobacteriaceae virulence factor. Several physicians carried virulence factor genes related to siderophore systems, P fimbriae, and type 1 fimbriae, which play an important role in facilitating extraintestinal infections by promoting iron acquisition and host-cell adhesion.^21^ Given the severe risks of *E. coli* infections in patients with cirrhosis^3^, these findings underscore the necessity of robust surveillance and infection prevention measures to minimize cross-colonization in healthcare settings.

This study included an analysis of paired physician and cirrhosis patient fecal samples collected at same two healthcare facilities within the United States. A limitation of this approach is the highly unequal sample sizes limiting statistical power, which partly reflects the feasibility of physician recruitment as well as the greater ratio of patients compared to physicians. Nonetheless, the proportion of detectable opportunistic pathogens (*Enterococcus, E. coli, K. pneumoniae*) and associated VFs was far greater in the cirrhosis patients compared to the paired physician samples. Several highly notable ARGs were also unique to the cirrhosis cohort, including *bla*TEM, *bla*CTX, and quinolone resistance genes. Further comparison with a larger cohort of physicians in the same country but not interacting with these patients indicated a decrease in ARG richness in cirrhosis patients. This observation is likely attributed to decreased taxonomic diversity in the gut microbiome. We identified that the *tetA*(46) gene was significantly enriched in the decompensated patient population, which was detected on *Streptococcus* MAGs. This aligns with prior work reporting *Streptococcus* enrichment in gut microbiome of cirrhosis patients driven by translocation of oral microbes.^22^

Many clinically important ARGs in Enterobacteriaceae are often co-encoded on resistance plasmids, which can be horizontally transferred across Enterobacteriaceae species. ^23^While short-read shotgun metagenomic sequencing is useful for overall characterization of ARG and VF profiles in fecal samples, short-read sequencing provides limited resolution for identification and assignment of mobile ARGs to microbial hosts.^24^ Screening of contigs still showed several plasmid replicons were co-encoded on contigs with ARGs, including aminoglycoside, quinolone, and β-lactam resistance determinants. Additionally, we identified a decompensated cirrhosis patient co-encoding siderophore (*iutABCD)* genes and plasmid replicon on the same contigs. This approach is likely to represent a fraction of the mobile traits as plasmids frequently assemble into fragmented contigs that cannot be fully resolved or linked to specific microbial genomes without long-read scaffolding or proximity ligation techniques.^5,24^ Nonetheless, these results suggest that further study to identify plasmid transmission between patients or to healthcare workers is warranted.

In addition, the study is limited by the low number of physician participants, self-completed questionnaires pertaining to exposures and outcomes, and a large patient to physician ratio in the Virginia cohort. Despite these limitations, the differences across country types and exposures are informative and represent a step forward in cirrhosis infection pathophysiology.

Collectively, our findings indicate that a cohort of globally obtained physician samples showed variations in clinically relevant ARGs driven by underlying microbial diversity which was higher in LMICs. Most clinically relevant ARGs and VFs in cirrhosis patients were not found in physicians. While most physicians are unlikely to represent primary reservoirs of clinically relevant ARGs of VFs, individual physicians could still play a role in bidirectional transmission in healthcare settings. Continued global surveillance of antimicrobial resistance is needed to better understand the prevalence, emergence, or transmission of drug-resistant pathogens in healthcare settings.

## METHODS

### Sample Collection and Processing:

Physicians from across the world were invited to participate in this study using individual contact. Those who expressed interest were provided pre-addressed fecal collection kits with DNA/RNA Shield and instructions for sample collection using Zymo kit. Consent was provided verbally as approved by the Richmond VA IRB protocol. The physicians were then free to either return the kit or not based on their preference. Those who agreed verbally were sent a blank excel database to provide deidentified clinical and demographic information linked only to the barcode on the tube where samples were collected. The barcodes of the kits were not linked to individual physicians and were not noted at the time of dispensing kits to avoid inadvertent identification. The data inquired in the excel sheet included demographics, region of practice, years and details of their practice (inpatient, outpatient, intensive care unit, number of patients seen), number of patients with resistant infections, recent antibiotic use, and travel. Physicians who reported more than 10 inpatients or outpatients per week were classified as having high exposure for the respective variables. Physicians who reported contact with more than five patients with resistant infections per month were classified as having high exposure levels.

A cohort of outpatients with cirrhosis were enrolled prospectively concurrent to the physician stool collection. The protocol was approved by the Richmond VA IRB and all subjects provided consent before study initiation. For these patients, cirrhosis was diagnosed using liver biopsy, or history of or current decompensation (variceal bleeding, hepatic encephalopathy, ascites), or presence of varices in the setting of chronic liver disease, or transient elastography > 12.5KPa. Cirrhosis patients were divided into compensated and decompensated based on established definitions^25^. All collections were performed between 2021 and 2023. The Richmond VA IRB operates in accordance with the Declaration of Helsinki.

DNA extraction was performed at George Mason University using the DNeasy Blood & Tissue Kits (Qiagen). Library preparation and sequencing was performed at the University of Minnesota Genomics Center (UMGC) using a standardized institutional workflow. In brief, DNA concentration and quality were assessed for all samples using a fluorometric PicoGreen assay and a NanoDrop spectrophotometer. Library preparation was performed using the Illumina Nextera XT DNA Sample Preparation Kit (Cat. No. FC-131-1096). Pool libraries were subsequently sequenced on an AVITI24 instrument (Element Biosciences, San Diego, USA), with a median raw read depth of 31 million reads for physician samples and 19 million reads for cirrhosis patient samples.

### Metagenomic Sequencing and Analysis:

Demultiplexed shotgun metagenomic reads were processed using KneadData (v0.12.3) to remove adapter sequences, low-quality reads, and human-derived contaminant reads.^26-28^ Sequence quality was assessed using FastQC and compiled via MultiQC^29^. Microbial taxonomic profiling of samples was performed using MetaPhlAn 4 (v4.2.4; Database: mpa_vJan25_CHOCOPhlAnSGB_202503).^26^ ARG and VF identification was performed using KMA (v1.6.8) against the ResFinder (v2.6.0) and the VFDB (setA; downloaded March 2026) databases, respectively. ^30-33^Alignments were quality filtered to retain only hits with at least 90% adjusted coverage (*i.e.*, percent coverage multiplied by the percent identity against reference template). ARG and VF reads were normalized as fragment counts per kilobase per genome equivalent (RPKG), which accounts for gene length and sequencing depth using MicrobeCensus (v1.1.0) estimated genome equivalents.^34^

A parallel assembly-based approach was implemented for the taxonomic linkage of ARGs to microbial metagenome-assembled-genomes (MAGs). Quality-filtered reads were assembled into contigs using MEGAHIT (v1.2.9, minimal contig length 500 bp) and then binned into putative metagenome assembled genomes using METABAT (v2:2.17, minimal contig length 2500bp).^35,36^ MAG quality was assessed using CheckM (v1.2.4) and filtered to retain MAGs with ≥ 85% completeness and ≤ 5% contamination.^37^ Taxonomic classification of MAGs was performed using GTDB-Tk (v2.6.1; Database: release226) with the classify_wf workflow^38^. All contig sequences were screened against ResFinder, VFDB, and PlasmidFinder databases and high-quality MAGs were screened against the ResFinder database with Abricate (v1.2.0)^31,33,39^.

Prevalence differences between groups were assessed using a Fisher’s exact test in R (v4.6.1). Global differences in abundance were assessed using a Kruskal-Wallis test; post-hoc analysis was performed using a Dunn test. Resulting p-values were adjusted using a Benjamin-Hochberg (BH) correction. The association of ARG diversity with species richness and country income were assessed using a generalized linear model with a Poisson distribution and log link function. Plots were constructed in R using the ggplot2 (v4.0.3) and Pheatmap (v1.0.13) packages.

## Supplementary Material

This is a list of supplementary files associated with this preprint. Click to download.

• SupplementPoopyDoc09182026NPJ.docx

## Figures and Tables

**Figure 1:**
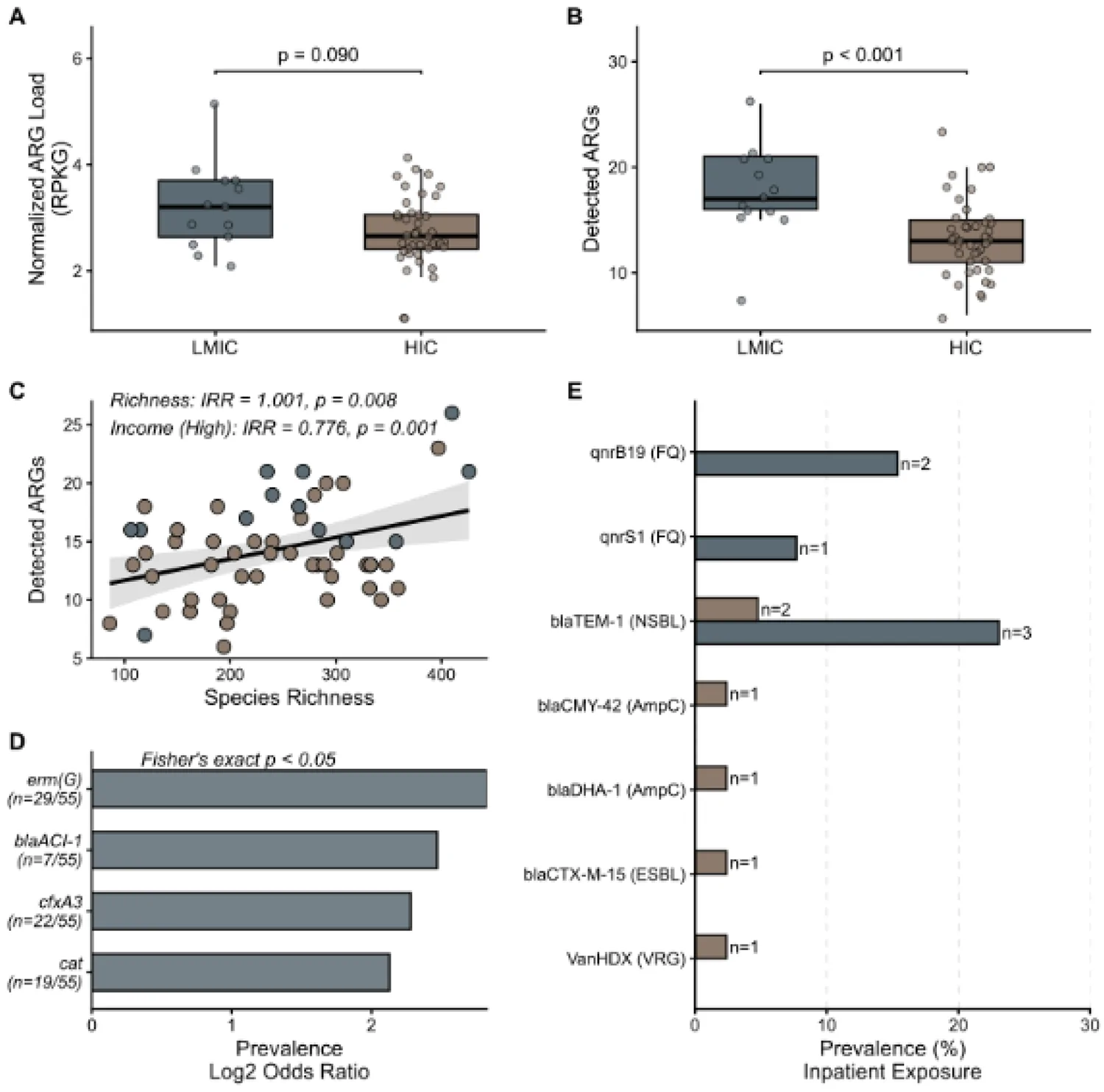
ARG carriage by physicians, stratified by practicing-country income level. (**A**) Comparison of the total ARGs load, normalized as reads per kilobase per genome (RPKG). (**B**) Comparison of detected ARG richness between physicians practicing in HICs and LMICs. (**C**) Scatterplot showing the association between microbial species richness and number of detectable ARGs within each sample. (**D**) Odds ratios from Fisher’s exact tests for ARGs with higher prevalence in LMICs compared with HICs (nominal p < 0.05). (**E**) Prevalence of clinically-relevant ARGs carried by physicians in LMICs and HICs. The resistance class is shown next to each gene (FQ, fluoroquinolone resistance; VRG, vancomycin resistance gene; ESBL, extended-spectrum β-lactamase; AmpC, AmpC β-lactamase; NSBL, narrow-spectrum β-lactamase).

**Figure 2:**
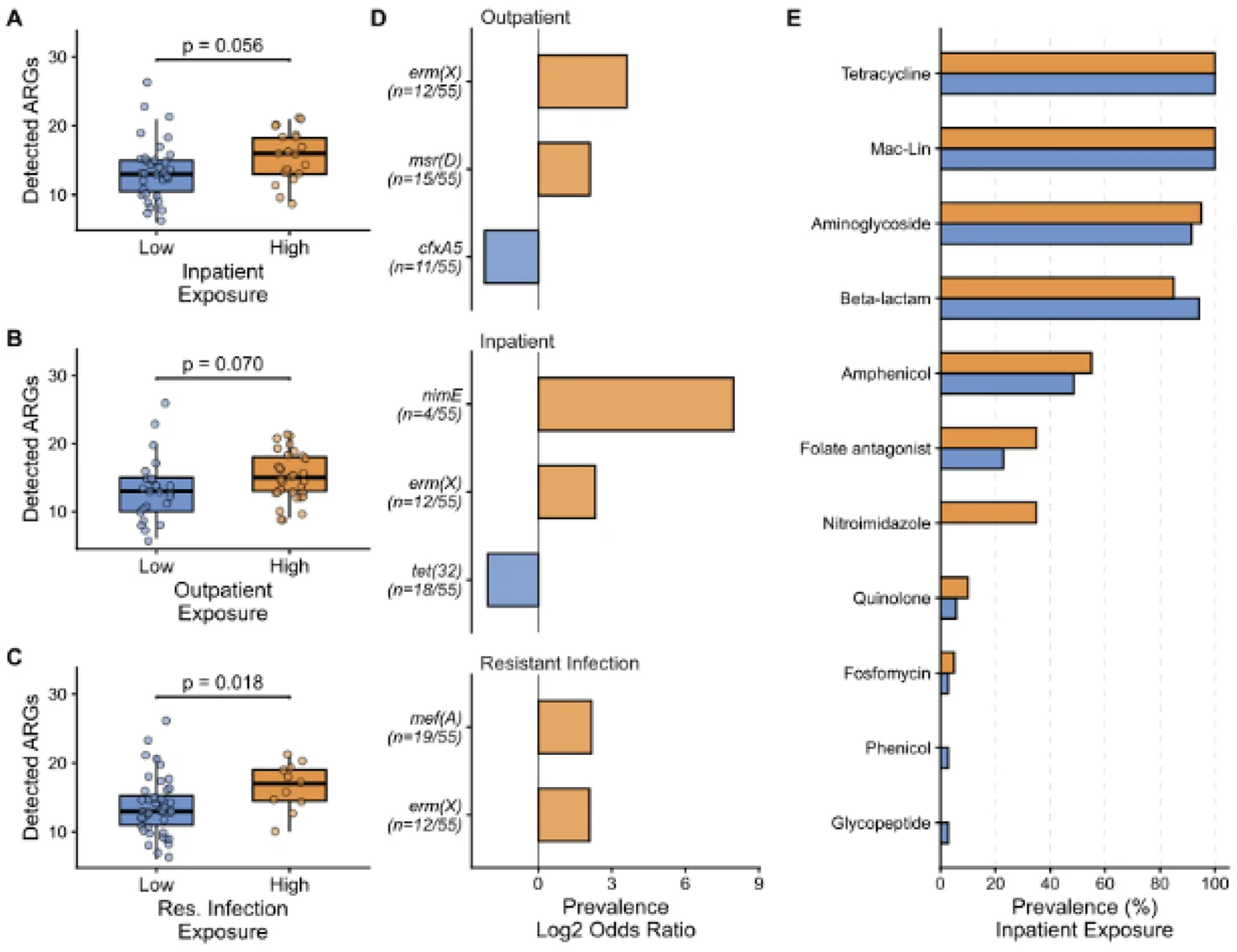
ARG carriage by physicians, stratified by cirrhosis patient exposure. (**A-C)** Comparison of the detected ARG counts (richness) across physicians with low and high levels of exposure to outpatients (**A**), inpatients (**B**), and patients with known resistant infections (**C**). Odds ratios from Fisher’s exact tests for ARGs with differential prevalence between exposure groups (nominal p < 0.05). (**E**) Prevalence of detected ARG classes across doctors stratified by low and high levels of exposure to cirrhosis inpatients. Orange bars represent high exposure; blue bars represent low exposure.

**Figure 3:**
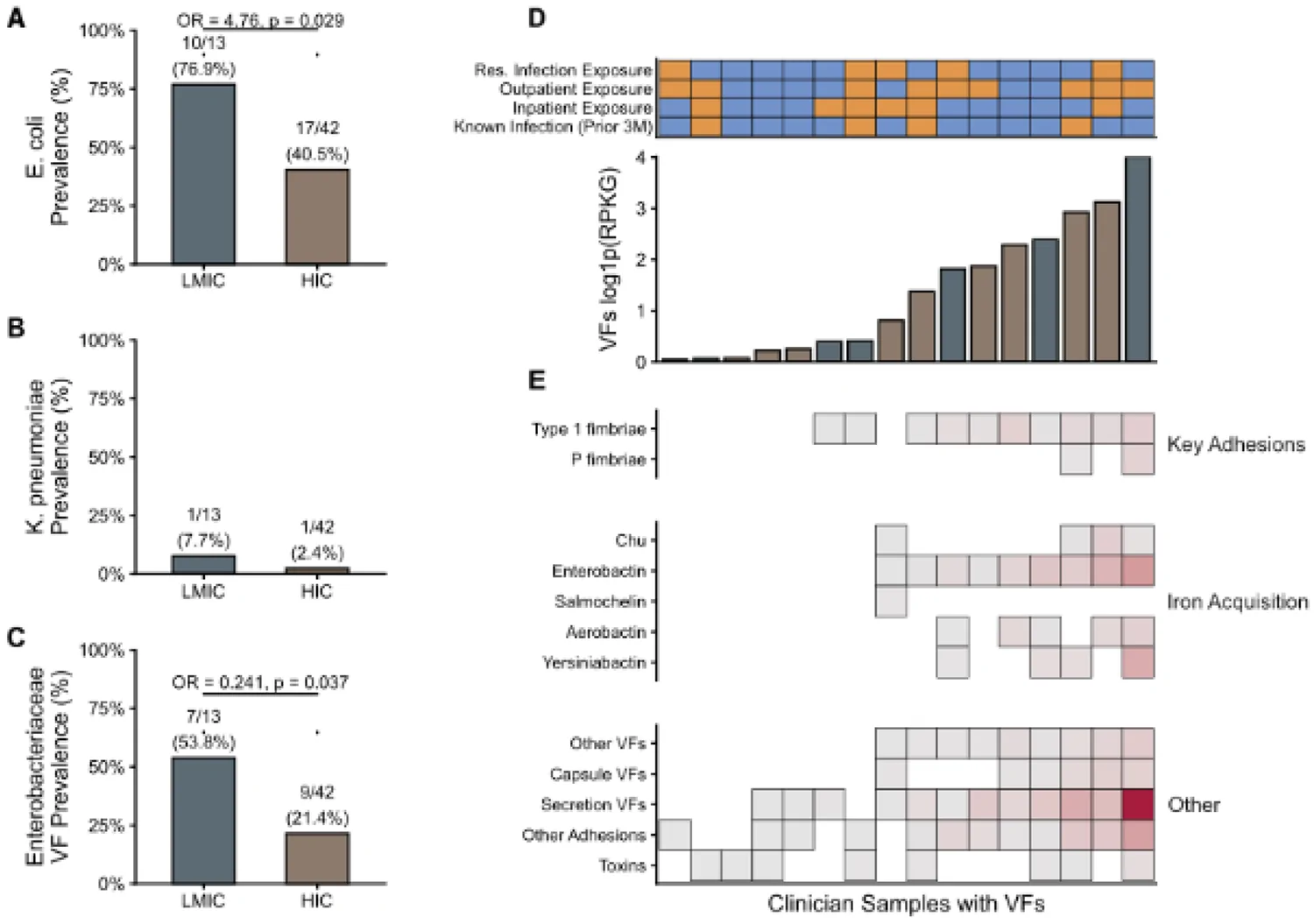
Carriage of Enterobacteriaceae and associated virulence factors by physicians. (**A**) Prevalence of detectable *E. coli* carriage among physicians from LMICs. (**B**) Prevalence of detectable *K. pneumoniae* carriage among physicians. (**C**) Prevalence of detectable Enterobacteriaceae-associated VFs carriage among physicians. (**D**) Total normalized abundances of Enterobacteriaceae-associated VFs across physicians. Bars represent individual physician samples with detectable VFs, colored by income status for country of practice. Heatmap above shows physician demographics, with orange indicating a high level of cirrhosis patient exposure or known recent infections. (**E**) Heatmap of normalized VFs across physician samples, grouped by VF function. Color intensity reflects normalized RPKG within each sample, with red indicating higher normalized abundances.

**Figure 4:**
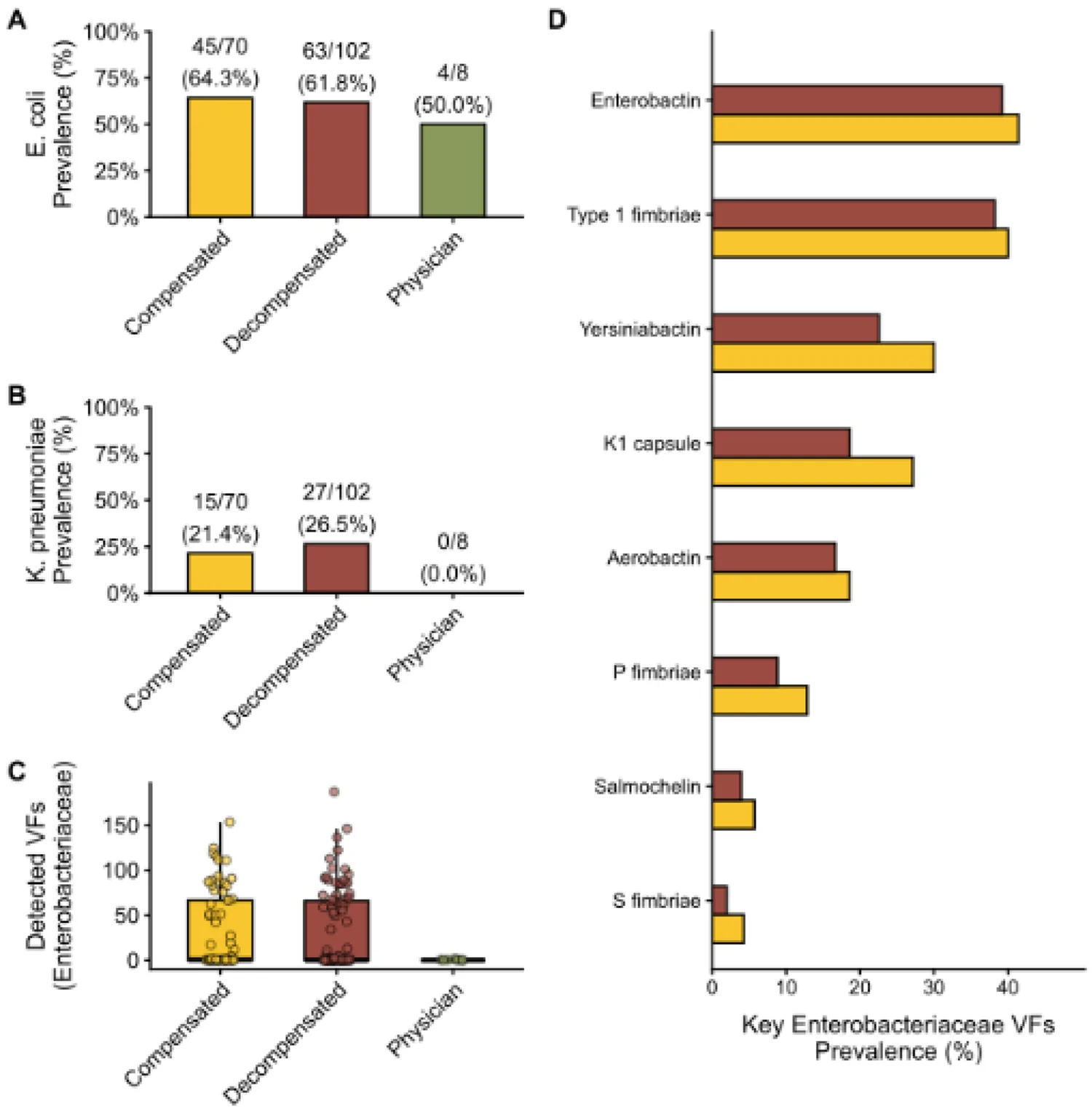
Comparison of VFs carried by physicians and cirrhosis patients in same US medical centers. (**A**) Prevalence of detectable *E. coli* carriage in physicians and patients with cirrhosis. (**B**) Prevalence of detectable *K. pneumoniae*carriage in physicians and patients with cirrhosis. (**C**) Prevalence of detectable Enterobacteriaceae-associated VFs carriage in physicians and patients with cirrhosis. (**D**) Prevalence of key clinically relevant VFs unique to patients with cirrhosis. Yellow indicates compensated cirrhosis samples; red indicates decompensated cirrhosis samples.

**Figure 5:**
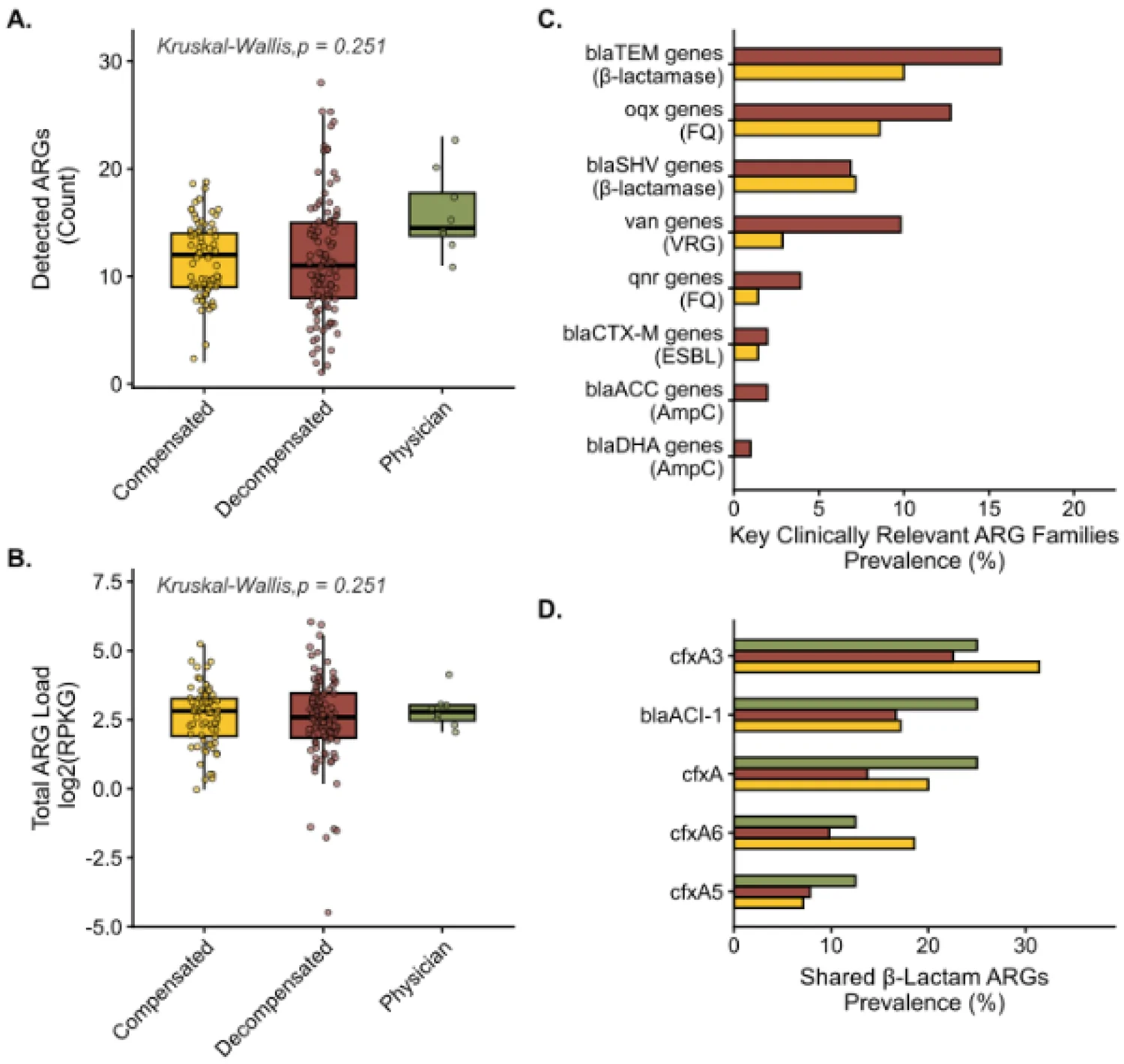
Comparison of ARGs carried by physicians and cirrhosis patients in same US medical centers. (**A**) Comparison of detected ARG richness between physicians and patients with cirrhosis. (**B**) Comparison of the total ARGs load, normalized as reads per kilobase per genome (RPKG). (**C**) Prevalence of key clinically relevant ARGs unique to patients with cirrhosis, grouped by gene family. (**D**) Prevalence of β-lactam resistance genes detected in both physicians and patients with cirrhosis.

## Data Availability

Host-removed sequence reads for physician fecal metagenomes are available on the NCBI Sequence Read Archive (SRA) under NCBI BioProject PRJNA1521878. Sequence reads for patients are available on the NCBI Sequence Read Archive (SRA) under PRJNA1521590. Reviewer Link: https://dataview.ncbi.nlm.nih.gov/object/PRJNA1521878?reviewer=2nftv1vk5ate3jr4husolmcm8c
